# Transpositionally active episomal *hAT *elements

**DOI:** 10.1186/1471-2199-10-108

**Published:** 2009-12-14

**Authors:** David A O'Brochta, Christina D Stosic, Kristina Pilitt, Ramanand A Subramanian, Robert H Hice, Peter W Atkinson

**Affiliations:** 1 Center for Biosystems Research, University of Maryland Biotechnology Institute, 9600 Gudelsky Drive, Rockville, MD 20850, USA; 2 Department of Entomology, University of California, 900 University Avenue, Riverside, CA 92521, USA; 3 Institute for Integrative Genome Biology, University of California, 900 University Avenue, Riverside, CA 92521, USA

## Abstract

**Background:**

*hAT *elements and V(D)J recombination may have evolved from a common ancestral transposable element system. Extrachromosomal, circular forms of transposable elements (referred to here as episomal forms) have been reported yet their biological significance remains unknown. V(D)J signal joints, which resemble episomal transposable elements, have been considered non-recombinogenic products of V(D)J recombination and a safe way to dispose of excised chromosomal sequences. V(D)J signal joints can, however, participate in recombination reactions and the purpose of this study was to determine if *hobo *and *Hermes *episomal elements are also recombinogenic.

**Results:**

Up to 50% of *hobo/Hermes *episomes contained two intact, inverted-terminal repeats and 86% of these contained from 1-1000 bp of intercalary DNA. Episomal *hobo/Hermes *elements were recovered from *Musca domestica *(a natural host of *Hermes*), *Drosophila melanogaster *(a natural host of *hobo*) and transgenic *Drosophila melanogaster *and *Aedes aegypti *(with autonomous *Hermes *elements). Episomal *Hermes *elements were recovered from unfertilized eggs of *M. domestica *and *D. melanogaster *demonstrating their potential for extrachromosomal, maternal transmission. Reintegration of episomal *Hermes *elements was observed *in vitro *and *in vivo *and the presence of *Hermes *episomes resulted in lower rates of canonical *Hermes *transposition *in vivo*.

**Conclusion:**

Episomal *hobo*/*Hermes *elements are common products of element excision and can be maternally transmitted. Episomal forms of *Hermes *are capable of integration and also of influencing the transposition of canonical elements suggesting biological roles for these extrachromosomal elements in element transmission and regulation.

## Background

Transposons are ancient and ubiquitous inhabitants of genomes that have played a significant role in genome evolution across kingdoms [1]. Their activity has played a notable role in genome expansions (e.g. *Zea maize, Aedes aegypti*) and has contributed in a variety of ways to the generation of variation within genomes that subsequently has been subjected to natural selection during evolution [2]. Whole-genome sequence analysis has contributed greatly to revealing how the domestication of transposons has contributed to genome expansion and complexity [3]. These studies have shown that transposons belonging to the *hAT*, *piggyBac, mariner-Tc1 *and *Harbinger *superfamilies have persisted in eukaryote genomes not only as mobile DNA but as genes with new functions [3-6]. Indeed domestication of transposons is now seen, along with low frequency mobility of active transposons, as a viable strategy for the long-term persistence of these sequences in genomes. A notable example of domesticated transposable elements contributing to genome evolution is the somatic gene rearrangement system (V(D)J recombination) that leads to the generation of B- and T-cell antigen receptors in the adaptive immune system of vertebrates. V(D)J recombination is mediated by the transposase-like proteins RAG1/RAG2 and DNA recombination signal sequences (RSS) flanking different gene segments that serve the same function as the terminal inverted repeats of transposable elements [7]. The ancestral transposable elements from which the RAG/RSS system evolved have been proposed to be either *Transibs *[8] or *hAT *transposons [9]. The hypothesis that the V(D)J system has evolved from *Transibs *is based on structural similarities between RAG recombinases and *Transibs *transposases along with the fact that both systems result in 5 bp duplications following sequence integration [8,10,11]. The idea that the V(D)J recombination system and extant *hAT *transposons evolved from a common ancient recombination system is also based on structural similarities between the RAG recombinases and *hAT *transposases as well as mechanistic similarities between the corresponding recombination reactions. The most striking similarity is the formation of terminal hairpin structures on the DNA ends flanking the gap created following transposon and RSS excision.

V(D)J recombination involves the excision of RSS-flanked sequences resulting in the fusion of the terminal RSSes and the formation of covalently closed circular DNA (signal joints). Originally it was thought that signal joints were safe, inert byproducts of recombination in which the reactive 3' hydroxyls at the ends of the excised RSS were prevented from participating in subsequent recombination and eventually degraded [11,12]. It is now clear that this is not the inevitable fate of these episomal molecules. Signal joints have been shown to be capable of reintegrating both *in vitro *and *in vivo *and recent studies have shown that they can contribute to genome instability and result in pathologies [10,11,13-15].

*hAT *element excision as well as the excision of transposable elements belonging to other classes and families of transposons can also lead to creation of covalently closed episomes [16-30]. While such forms of transposable elements have been recognized, their significance, if any, has been unclear. Although thought by some to be potential transposition intermediates, it is clear that for the *Hermes *transposon and probably all *hAT *elements, this is not the case [9]. Nonetheless, as recognized by Arca et al. "the widespread occurrence of extrachromosomal circles suggests that they may have a functional role in transposition, rather than being inactive byproducts"[29]. Kempken and Kück suggested that episomal forms of the *hAT *element *Restless *in the fungus *Tolypocladium inflatum *might facilitate horizontal transfer between nuclei in heterokaryons that form occasionally as a result of anastomoses of fungal hyphae [30]. However, this would require that episomal forms of transposable elements be capable of undergoing transposition and reintegratioin but, unfortunately, this has been rarely tested. Yang et al. reported data suggesting that reintegration of an episomal *Ds *element had occurred in *Arabidopsis*, however this appeared to have occurred via an illegitimate recombination event and not via canonical transposase/inverted-terminal repeat-mediated transposition [31]. The only report of an effort to empirically assess the transposition potential of an episomal eukaryote transposable element failed to find any evidence of episomal *Ac/Ds *reintegration in *Nicotiana tabacum *[17]. While Gorbunova and Levy concluded that episomal *hAT *elements are merely abortive excision products, the recent findings of V(D)J signal joint recombination activity [10,11,13-15] and the results reported here indicate that these elements are likely to have biological significance.

We describe here the biology of the episomal forms of the closely related and functionally interactive *hAT *elements *hobo *and *Hermes*. *hobo *was originally isolated from *D. melanogaster *[32] and *Hermes *was isolated from the housefly, *Musca domestica *[33]. The amino acid sequences of the transposases of these elements are 55% identical and the terminal inverted repeat sequences are also highly similar [34]. Because of these similarities, these elements are capable of interacting, resulting in cross-mobilization [33,35].

We show that the creation of episomal *hobo*/*Hermes *elements occurs frequently during element excision relative to the overall element excision rate. A large proportion of the episomal forms of these elements contained all of the molecular information required for transposition. Episomal *hAT *elements did not only arise during element excision in experimental systems but also in natural systems in which elements are normally undergoing transposition. Episomal forms of *Hermes *elements were detected in somatic tissue as well as unfertilized eggs of *Musca domestica *and transgenic *Drosophila melanogaster*. The detection and recovery of episomes from the somatic tissue of *M. domestica*, the natural host of *Hermes*, indicates that this element is active in this species. The presence of episomal *Hermes *in unfertilized eggs indicates that they can be transmitted maternally. We describe the reintegration of various forms of episomal *Hermes *elements *in vitro *and *in vivo *demonstrating that, like V(D)J signal joints, these molecules are recombinogenic and possibly able to contribute to the dynamics of transposable element transmission in nature. Finally, we show that the presence of episomal *Hermes *elements can influence the transposition of canonical *Hermes *elements suggesting that episomal elements may play a role in the regulation of element movement.

## Results

### *hobo *and *Hermes *episomes

*hobo *episomes were recovered during *in vivo hobo *mobility assays performed in developing *D. melanogaster *embryos. They were obtained following the simultaneous introduction into developing *D. melanogaster *of 'donor' plasmids containing a *hobo *element carrying a kanamycin resistance gene, an origin of replication and the *E. coli lacZ *alpha peptide coding region and 'helper' plasmids containing the *hobo *transposase open reading frame under the control of a heat-inducible promoter. Following recovery from developed embryos approximately 18 hours post-injection, recovered DNA was digested with restriction endonucleases *Kpn*I and *Xba*I. *hobo *episomes did not contain *Kpn*I and *Xba*I restriction sites and were resistant to digestion by these restriction endonucleases while donor and helper plasmids were cleaved multiple times. Introduction of the digested DNA into *E. coli *permitted the recovery of only episomal forms of *hobo*. *hobo *episomes were readily recovered under these conditions and for approximately every 1000 donor plasmids recovered from injected embryos six *hobo *episomes were isolated ( = 0.0061, *SEM *= 0.0040, *n *= 7)). *hobo *excision products consisting of donor plasmids with an empty *hobo *integration site were also recovered during the same experiments at a rate of approximately three in every 1000 recovered donor plasmids ( = 0.0031, *SEM *= 0.0006, *n *= 5). Analysis of variance indicated that there was no significant statistical difference between the frequency of recovery of *hobo *episomes and empty *hobo *donor sites (*F*_1,10 _= 0.3831, *P *= 0.5497).

A variety of specific forms of *hobo *episomes were recovered from plasmid-based mobility assays, differing in the sequence of the junction between the left and right terminal sequences (Table 1). Of the 38 episomes recovered from plasmid-injected embryos and whose sequence was determined, 15 (39%) contained intact left and right inverted terminal repeats and contained all of the information necessary for transposition. Three of these 15 'intact' episomes were perfect end-to-end joints of the left and right terminal inverted repeats. The remaining 12 'intact' episomes contained from 1 to 20 nucleotides between the inverted terminal repeats. Three of these episomes had intercalary DNA with sequences related to the inverted terminal repeats of *hobo *(ie. TTCTTCT, AGAACTTCTCTG, ATGCGGCTGCAGTTCTCTG). The remaining 23 (61%) 'defective' *hobo *episomes were missing one (19) or both (4) of the inverted terminal repeats and had variable amounts of their sub-terminal sequences deleted. These deletion-containing episomes are not expected to be transpositionally competent.

**Table 1:** Structure of *hobo *episomes from *D. melanogaster*

left ITR^*a*^	Intercalary DNA	right ITR^*a*^	**no.^b^**
→		←	3
→	T	←	5
→	A	←	3
→	AT	←	1
→	TTCTTCT^*c*^	←	1
→	AGAACTTCTCTG^*c*^	←	1
→	ATGCGGGCTGCAGTTCTCTG^*c*^	←	1
→		-187	1
→		-228	1
→		-235	1
→		-275	1
→		-364	1
→		-592	1
→	GG	-1	1
→	A	-19	1
→	ACAACGA	-12	1
-11		←	1
-93		←	2
-120		←	1
-125		←	1
-143		←	2
-198		←	1
-274		←	1
-294		←	1
-2		-4	1
-18		-6	1
-11	GCACAGTCAACGATCGCCGCA	-248	1
-43		-18	1

^*a*^arrows represent intact ITRs and their orientation. The size of deletions is indicated by negative numbers.

^*b*^number recovered.

^*c*^sequences related to hobo ITR (right ITR: TGCTGTTCTCTG)

**Figure 1:**
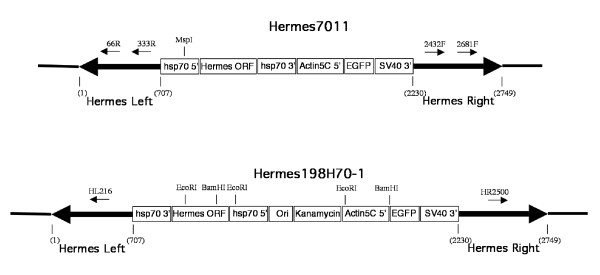
**Structure of the autonomous *Hermes *elements used in this study**. *Hermes *terminal sequences are shown as thick black arrows with associated nucleotide numbers in parenthesis. Primers used are thin arrows with their corresponding name. The position of relevant restriction endonuclease sites are shown using conventional abbreviations (BamHI, EcoRI, MspI). Hermes ORF - the complete *Hermes *transposase open reading frame. hsp70 5'-promoter from the *hsp70 *gene of *D. melanogaster*. hsp70 3' - 3' untranslated region of the *hsp70 *gene of *D. melanogaster*. Actin5C - promoter from the *Actin5C *gene of *D. melanogaster*. EGFP- the complete coding region for Enhanced Green Fluorescent Protein. SV40 3'- 3' untranslated region of Simian Virus 40. Ori - ColE 1 origin of replication. Kanamycin - kanamycin resistance gene.

*Hermes *episomes were recovered from two transgenic *D. melanogaster *containing integrated, self-mobilizable *Hermes *elements (*Hermes*7011 and *Hermes*198H70-1) (Figure 1). Episomes in insects containing *Hermes*7011 were detected using a nested PCR strategy using two pairs of *Hermes *terminus-specific primers oriented so that PCR products will only arise when the termini are joined end-to-end (see Methods, Figure 1). The DNA sequence was determined for a sample of the cloned products arising from this PCR reaction (Table 2). Thirty-five percent (9/26) of the episomes recovered using this method had intact inverted repeats and all of the sequence information necessary for transposition. Only two of the recovered episomes were perfect end-to-end joins. Thirty-one percent (8/26) had only intact left inverted terminal repeats with variable amounts of the right end being deleted while 19% (5/26) had a reciprocal structure with intact right inverted terminal repeats and variable amounts of the left end being deleted. Fifteen percent of the recovered episomes were missing both inverted terminal repeats and adjacent sub-terminal sequences.

**Table 2:** Structure of *Hermes *episomes from transgenic *D. melanogaster - PCR*

left ITR^*a*^	Intercalary DNA	right ITR^*a*^	no.^b^
**→**		←	2
→	A	←	5
→	ATAC	←	1
→	ACTAC	←	1
→		-3	1
→		-18	1
→		-22	1
→		-31	1
→		-42	1
→	A	-5	1
→	A	-10	1
→	ACAACGA	-12	1
-7		←	1
-60		←	1
-16	A	←	1
-21	T	←	1
-30	T	←	1
-14		-50	1
-17		-38	1
-36		-25	1
-43		-18	1

^*a*^arrows represent intact ITRs and their orientation. The size of deletions is indicated by negative numbers.

^*b*^number recovered

*Hermes *episomes were also recovered from transgenic *D. melanogaster *using a plasmid rescue strategy. Autonomous *Hermes *elements containing a kanamycin resistance gene and an origin of replication permitted *Hermes *episomes to function as replicons in *E. coli*. Sixty *Hermes *episomes were recovered from adults and larvae of four independent 'lines' of transgenic *D. melanogaster *(Table 3). Approximately 50% (29/60) contained 'intact' *Hermes *elements with complete inverted terminal repeats. Only three (5%) were perfect end-to-end joins of the inverted terminal repeats while the remaining 26 contained variable amounts of intervening nucleotide information ranging from 1 bp to 1 kb. All other episomes contained 'defective' *Hermes *elements lacking either one or both inverted terminal repeats with variable amounts of sub-terminal sequences deleted; 20% had intact left inverted terminal repeats and 25% had intact right inverted terminal repeats. *Hermes *episomes were also detected in unfertilized *D. melanogaster *eggs by PCR (Table 3). The structure of episomes found in unfertilized eggs was generally similar to those found in other life stages although episomes with only the right inverted repeat (53%) were recovered more frequently in unfertilized eggs than from adults and larvae (25%). Approximately half (8) of the episomes contained complete elements including perfect end-to-end joints and those with a small number of nucleotides between the inverted terminal repeats.

**Table 3:** Structure of *Hermes *episomes from transgenic *D. melanogaster - plasmid rescue*

left ITR^*a*^	Intercalary DNA	right ITR^*a*^	A^b^	B^*b*^	C^*b*^	D^*b*^	*l*^*b*^	e^*b*^
→		←				2	1	1
→	A	←	4					1
→	T	←	1	1	1			
→	G	←				1		
→	C	←					1	
→	AC	←		1				
→	GAT	←	1					
→	TTGC	←			1			
→	GTGG	←				1		
→	GTCT	←		1				4
→	AAAG	←		1				
→	GCGGT	←	1					
→	CCATAC	←				1		
→	AGGTTT	←		1				
→	ACTCAAC	←	1					2
→	GGCTGCAT	←			1			
→	CTCGGTACCAGATCTGCGG	←		1				
→	1 kb	←	1	3		1		
→		-3	1					1
→		-23		1				
→		-30	2					
→		-41					1	
→		-44			1			
→		-319		1				
→	T	-5				1		
→	T	-15			1			
→	G	-25			1			
→	A	-40		1				
→	GA	-16		1				
-1		←		1				
-6		←		1				
-12		←	1					
-14		←						1
-15		←				2		
-23		←			1			
-31		←				1		3
-79		←		1				3
-171		←					1	
-193		←		1				
-1	A	←						1
-4	T	←			1			
-5	G	←		1				
-10	T	←		1				
-11	C	←		1				
-14	T	←	1					2
-11		-44		1				
-248		-50	1					
-293		-115		1				
-1	T	-295		1				

^*a*^arrows represent intact ITRs and their orientation. The size of deletions is indicated by negative numbers.

^*b*^number recovered from adults of lines A,B,C,D, larvae (l, all lines) and unfertilized eggs (e).

*Hermes *episomes were also detected by PCR and recovered by plasmid rescue from transgenic *Aedes aegypti *containing the same autonomous *Hermes *element as was in the transgenic *D. melanogaster *described above (*Hermes*7011). Episomes were recovered from developing embryos, larvae and from adult ovarial tissue and resembled those recovered from *D. melanogaster *in their sequence (Table 4).

**Table 4:** Structure of *Hermes *episomes from transgenic *Ae. aegypti*.

left ITR^*a*^	Intercalary DNA	right ITR^*a*^	o^d^	l^*d*^	em^*d*^
→		←^*c*^	1		
→	A	←^*c*^	1		3
→	GTCT	←^*b, c*^	1	7	
-6		←^*c*^		1	
-14		←^*b*^			1
-79		←^*b, c*^	1		1
-11	C	←^*c*^			
-14	T	←^*c*^		3	

^*a*^arrows represent intact ITRs and their orientation. The sizes of deletions are indicated by a negative number.

^*b*^from plasmid rescue.

^*c*^from PCR.

^*d*^o, ovaries; l, larvae; em, embryos.

**Figure 2:**
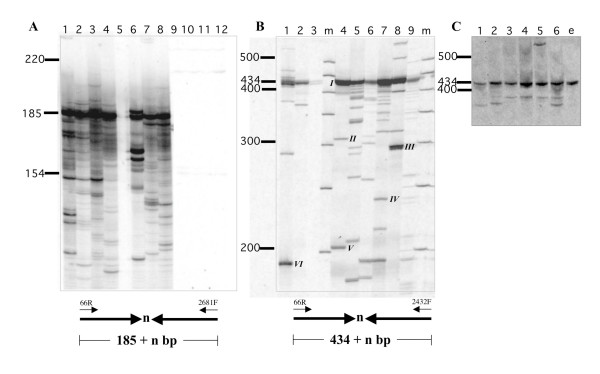
**Episomal detection using PCR**. **A**. PCR reaction products from 8 individual transgenic adult *D. melanogaster *(Canton S) with the autonomous element *Hermes *7011 (lanes 1-8). Lanes 9-12 contain the products of identical PCR reactions using the equal amounts of genomic DNA from non-transgenic Canton S individuals. The diagram below schematically illustrates the structure and size of PCR products arising from *Hermes *elements with their terminal inverted repeats (thick arrows) joined as shown using the primers indicated (66R, 2681F) with variable (n) numbers of nucleotides of intercalary DNA. The positions and size in basepairs of molecular weight standards are indicated. **B**. PCR reaction products from 9 individual *M. domestica *adults from 3 geographically distinct natural populations. The diagram below schematically illustrates the structure and size of PCR products arising from *Hermes *elements with their terminal inverted repeats (thick arrows) joined as shown using the primers indicated (66R, 2431F) with variable (n) numbers of nucleotides of intercalary DNA. Molecular weight markers (m) and their sizes in basepairs are shown. Roman numerals refer to bands that were excised, reamplified, cloned and sequenced. The results are shown in Table 5. **C**. PCR reaction products from 6 individual *M. domestica *adults from the laboratory colony, Cs. DNA from unfertilized eggs (e) from this colony was also used as template in these PCR reactions. The positions and size in basepairs of molecular weight standards are indicated.

*Hermes *is a natural inhabitant of the genome of *M. domestica *and has been detected in all individuals (n = 65) sampled from 13 populations from four continents [36]. Because the *Hermes *element in these populations did not naturally contain a functional origin of replication, episomes could not be recovered by plasmid rescue and could only be detected using a PCR-based method that relied on the use of PCR primers specific to the right and left terminal sequences of the element that would result in PCR products only when the termini were joined end to end. Total genomic DNA isolated from individual *M. domestica *from three populations had evidence of episomal *Hermes *elements as indicated by the recovery of PCR products using end-specific primers (Figure 2B). The most abundant forms were perfect (434 bp) or near perfect (> 434 bp) end-to-end joints containing all of the information necessary for transposition (Figure 2B; Table 5). As in transgenic *D. melanogaster*, 'defective' forms were also recovered with variable amounts of terminal sequences deleted from PCR products less than 434 bp (Figure 2B). All 'defective' forms recovered were missing the right inverted terminal repeat and, in one case, both termini were absent (Table 5). Finally, episomal *Hermes *elements were detected in unfertilized eggs of *M. domestica *(Figure 2C).

**Table 5:** Structure of *Hermes *episomes from *M. domestica*.

left ITR^*a*^	Intercalary DNA	right ITR^*a*^	
→		←	^*I*^
→	A	←	^*I*^
→	ATAC	←	^*I*^
→	ACTAC	←	^*I*^
→	CGTTTTCCAC	←	
→		-34	
→		-69	
→	G	-106	
→		-116	
→	A	-125	^*II*^
→		-129	
→	G	-134	
→		-138	^*III*^
→		-144	
→	TT	-178	
→		-195	^*IV*^
→	G	-228	
→		-231	^*V*^
→		-240	
→		-241	
→		-243	
→	A	-259	
→		-264	
→	G	-269	
→		-272	
→		-308	
-34		-113	

^*a*^arrows represent intact ITRs and their orientation. The size of deletions is indicated by negative numbers. Roman numbers refer to bands in Figure 2B.

### Re-integration of *Hermes *episomes *in vitro*

**Figure 3:**
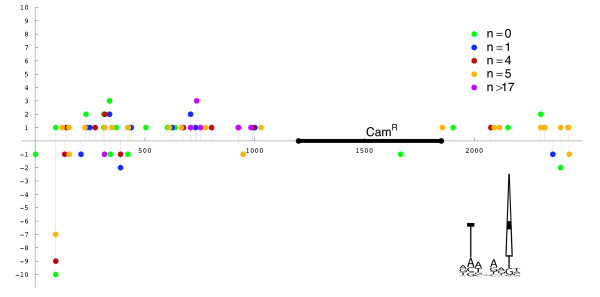
**Map of episomal *Hermes *integration sites in the target plasmid pGDV1**. The x axis refers to the linear form of the target plasmid and the position of the chloramphenicol resistance gene (CamR) is shown. The numbers on the x-axis refer to the length of the plasmid in basepairs. The y-axis is the number of integrations. Positive and negative values refer to elements that integrated into the target in opposite orientations. Five different classes of *Hermes *episomes were tested for their ability to integrate with each differing only in the amount of intercalary DNA (n) located between the inverted terminal repeats. The unweighted consensus sequence of the *Hermes *target site is graphically represented with a sequence logo [58] constructed with WebLogo [59].

The recombination potential of a variety of *Hermes *episomes was tested directly using a cell free *Hermes *transposition assay with purified *Hermes *transposase, episomal *Hermes *elements and a target plasmid. *Hermes *episomes with *n *nucleotides of intercalary DNA between the inverted repeats were tested where *n *was 0, 1, 4, 5, 17, 37,69 and 120 nucleotides. All episomal forms of *Hermes *tested were capable of transposition resulting in canonical 8 bp target site duplications with the sequence nTnnnnAn (Table 6 and Figure 3). The distribution of integration events within the target indicated that certain sites were preferred as we have previously described (Figure 3) although the apparent hot spots of integration *in vitro *are not the same as reported *in vivo *(Table 6) [37,38]. Integrations at nucleotide 94 of the target plasmid were recovered frequently however it is not known whether the primary nucleotide sequence of this target site (ATTGAGAT) is the major determinant of this site's preferred status.

**Table 6:** Integration of *Hermes *episomes into target DNA *in vitro*.

**Site^a^**	**N^b^**	Orientation^c^	Target^d^	Site^a^	N^b^	Orientation^c^	Target^d^
		**n = 0^5^**				**n = 1^5^**	
2	1	-	TTATAAAA	209	1	-	TTAAGTGG
94	1	-	ATTGAGAT	389	1	-	GTTTATAT
343	1	-	GGATATAT	2358	1	-	ATTCAGAG
423	1	-	GTTGGGAT	232	1	+	CCCTCAAC
1665	1	-	TTACCAAT	249	1	+	GTTTTGAT
2394	2	-	GTATGTAC	339	2	+	TTTGGGAT
94	1	+	ATTGAGAT	431	1	+	AGACGTAA
232	1	+	CCCTCAAC	438	1	+	ATATGCGT
313	2	+	TATGAGTA	624	1	+	GTCGTAAT
339	1	+	TTTGGGAT	708	1	+	GTAAAGAT
370	1	+	TAAAGCAC	731	1	+	GTTATGTC
423	1	+	GTTGGGAT	991	1	+	TTTAAAAG
505	1	+	TTATCGAC	1001	1	+	AATGAAAA
603	1	+	TTAAGAAA	2321	1	+	ATTGGAAT
638	1	+	CCAGAAAC				
1904	1	+	ATAGCAAC				
2154	1	+	GTATGCAC				
2303	1	+	GTTCCGAC				
		**n = 4**^5^				**n = 5**^5^	
94	2	-	ATTGAGAT	94	7	-	ATTGAGAT
135	1	-	ATGCAAAT	156	1	-	TGGAAAAT
389	1	-	GTTTATAT	948	1	-	TTGGAGAT
144	1	+	ATTCAAAT	2433	1	-	GATAATAC
274	1	+	GAATTTGA	123	1	+	ATAAAAAC
318	1	+	GTACAGAG	154	1	+	TTTGGAAA
676	1	+	ATTTATTA	227	1	+	ACCACCCC
804	1	+	TTGATGAT	318	1	+	GTACAGAG
925	1	+	ATATAAAT	350	1	+	TTACAAAC
990	1	+	CTTTAAAA	425	1	+	TGGGATAG
2076	1	+	GAGACATT	612	1	+	CTATTTTT
2428	1	+	TACCAGAT	666	1	+	ACAGTGAT
				710	1	+	AAAGATAA
		**n = 17**^5^		755	1	+	GTAGAAAT
927	1	+	ATAAATAC	777	1	+	ATATGGTT
708	1	+	GTAAAGAT	1029	1	+	ATTTTTGG
				1855	1	+	GAAGTGAA
		**n = 37**^5^		2091	1	+	CCCATCAA
753	1	+	GTGTAGAA	2115	1	+	AGCAACAA
				2303	1	+	GTTCCGAC
		**n = 69**^5^		2322	1	+	TTGGAATA
316	1	-	GAGTACAG	2394	1	+	GTATGTAC
				2429	1	+	ACCAGATA
		**n = 120**^5^					
736	3	+	GTCTGAAC				
983	1	+	AAATCGAC				

^1"^Site" of integration; nucleotide number of the first base of the target sequence.

^2^Number of occurrences observed.

^3^"+" refers to <left-*Hermes*-right> relative to target sequence; "-" is <right-left>

^4^"Target" is the first 8 bp following the right ITR and is written 5'-3'

^5 ^number of nucleotides separating the left and right ITRs of *Hermes*.

### Re-integration of *Hermes *episomes *in vivo*

Efforts to create transgenic *D. melanogaster *using *Hermes *gene vectors constructed from episomal forms of the element ('episomal *Hermes *gene vectors') resulted in the successful creation of transgenic insects in some cases. Germ-line transformation experiments were performed using well-established protocols (see Methods) involving the co-injection of vector and transposase-expressing helper plasmids into preblastoderm embryos followed by screening for expression of the dominant visible genetic marker present on the vector in the next generation. Episomal *Hermes *gene vectors containing 0, 17 and 37 bp of intercalary DNA separating the inverted terminal repeats did not result in the recovery of germ-line transformation events (n = 303; Table 7). On the other hand, identical episomal *Hermes *gene vectors with 80 bp and 160 bp of intercalary DNA separating the inverted terminal repeats did result in germ-line transformation in 22% (n = 23) and 28% (n = 54) of the germ-lines tested (Table 7). Integrated *Hermes *elements from these transgenic insects were analyzed and in all cases (n = 16) the *Hermes *element was found precisely inserted into *D. melanogaster *genomic DNA (Table 8). The 8 bp immediately flanking the left inverted terminal inverted repeat of the integrated *Hermes *elements conformed to the known target site consensus sequence of this element (Table 8) [37,38]. While we were unable to detect integration of episomal *Hermes *gene vectors with 0 bp or 17 bp of intercalary DNA, we discovered that these forms of the element actually inhibited transposition of canonical *Hermes *elements, i.e. elements with typical spacing of the inverted terminal repeats. Germ-line transformation experiments involving the co-injection of episomal *Hermes *gene vectors with 0 bp or 17 bp of intercalary DNA and canonical *Hermes *gene vectors (HermesActin5CEGFP or Hermes3 × P3EGFP which both contain over 2 kb of intercalary DNA and are effective gene vectors) failed to produce any transgenic insects (n = 306). Identical experiments using only the canonical *Hermes *gene vectors confirmed their functionality and transformation was observed at a frequency of approximately 10% (n = 49; Table 7).

**Table 7:** *In vivo *integration activity of episomal *Hermes *element in *D. melanogaster*^*a*^

Episomal Vector	Canonical Vector	Fertile G_0_s	G_0_s Producing Transgenics	Transformation Frequency
→ 0 ←		136	0	0
→ 17 ←		87	0	0
→ 37 ←		80	0	0
→ 80 ←		23	5	0.22
→ 160 ←		54	15	0.28
	*Hermes*Act5CEGFP	49	5	0.1
→ 0 ←	*Hermes*Act5CEGFP	81	0	0
→ 0 ←	*Hermes*3× P3EGFP	141	0	0
→ 17 ←	*Hermes*Act5CEGFP	84	0	0

^*a*^individual plasmids were each at 0.25 mg/ml during injection

**Table 8:** Target-site analysis of integrated episomal *Hermes *elements in *D. melanogaster*

Element^*a*^	8 bp Target^*b*^	bp flanking DNA^*c*^
*input vector*	CAGCCTGA	
80-1	ATGGCCAC	48
160-1	GTGTGAAC	210
160-2	GTGACTAT	94
160-3	AACTCCAC	195
160-4	GTGTAGGC	77
160-5	ATACGGAT	47
160-6	GTGTACAC	36
160-7	ATCCGTAT	25
160-8	GTGTCAAC	287
160-9	CCACACCC	118
160-10	GTGTGAGT	18
160-11	CTCTACAC	207
160-12	GTTTGCAG	28
160-13	ATTCACAT	232
160-14	ATGCGGAC	286
160-15	GTCCGGNN	5
consensus	NTNNNNAN	

^*a*^input vector was used for transformation; numbers refer elements from transgenic lines.

^*b*^8 bp immediately flanking the left inverted terminal repeat of *Hermes*.

^*c*^bp of DNA recovered from integrate element

## Discussion

Episomal forms of eukaryote transposable elements are somewhat of a conundrum in that they are characteristically associated with many transposable element systems but are of unknown biological significance [16-30]. Reports of V(D)J signal joints being recombinogenic and capable of reintegrating into the genome of the host suggest that perhaps episomal forms of transposable elements were also capable of reintegration [10,11,13-15].

*Hermes *and *hobo *episomes were detected and recovered in this study under a variety of conditions using both bacterial replicon recovery methods (plasmid-rescue) and PCR-based methods. PCR detection of episomal elements depended upon the juxtaposition of the left and right inverted terminal repeats. Integrated *hAT *elements arranged in tandem could also yield PCR products with a structure and sequence similar to episomes. It is unlikely, however, that such elements were responsible for the PCR products recovered in this study because of the diversity of products recovered. Two elements arranged in tandem are expected to yield an invariant PCR amplification product but such was not the case in this study where episomal forms of the elements were recovered that had varying numbers of nucleotides between the inverted terminal repeats. In *M. domestica *and transgenic *D. melanogaster*, 27 and 21 distinct episomal forms of *Hermes *were detected by PCR, respectively. The copy number of *Hermes *in each of these species was estimated to be less than ten making it unlikely that chromosomal elements arrayed in tandem were the templates yielding the PCR products recovered in this study [39]. Finally, plasmid rescue experiments that did not involve PCR resulted in the recovery of *Hermes *episomes with structures identical to those recovered by PCR analysis. Therefore, we found no evidence to support a conclusion other than that the DNA molecules described here were episomes of *hobo *and *Hermes*.

*Hermes *and *hobo *episomes appear to be abundant products of *hAT *element excision/transposition reactions under some conditions. *hAT *elements transpose by a cut-and-paste type mechanism that is initiated by excision of the element from a donor site [40]. The excised element and associated transposase form a synaptic complex that associates with a target molecule and integrates [40]. In interplasmid *hobo *mobility assays performed in insect embryos, element excision products (empty donor sites) and episomal forms of the element were recovered from the same reactions at frequencies that were not significantly different. Under the conditions used here episome formation occurred readily during *hobo *movement.

While episomal forms of *hAT *elements are prevalent, their structures are very diverse. Very few of the episomal forms of *Hermes *and *hobo *were precise end-to-end joints of the inverted terminal repeats although 40%-50% of the episomes recovered in this study contained both copies of the element's terminal sequences. The intercalary DNA between the element's inverted terminal repeats varied in both quantity and sequence with no single form being dominant. While the origin of intercalary DNA in most cases was unknown, in three of the *hobo *episomes recovered the intercalary DNA appeared clearly related to the inverted terminal repeat sequences of *hobo *(Table 1). These sequences appear to have arisen following the resolution of terminal hairpin structures prior to the formation of the episome. This is unexpected because hairpin structures are not usually formed at the termini of excised *hAT *elements but on the ends of the donor sequences instead [9]. These unexpected and unusual intercalary sequences associated with *hobo *episomes are not explained by our current models of *hAT *element transposition [9].

The most significant finding of this study is that episomal *hAT *elements can reintegrate into target DNA molecules resulting in canonical integration events both *in vitro *and *in vivo*. *Hermes *episomes with 0 - 17 bp of intercalary DNA between the terminal inverted repeats could integrate into DNA target molecules *in vitro*. In these studies precise quantitation of integration activity was not performed however recovery of the events analyzed in this study required extensive screening of target molecules recovered from multiple *in vitro *transposition reactions. *In vivo*, the integration of *Hermes *episomes with 0-37 bp of intercalary DNA separating the terminal inverted repeats was undetectable under the conditions used in these experiments and screening the progeny of a combined 303 fertile G_0_s. When the amount of intercalary DNA separating the terminal inverted repeats was 80 bp or greater the frequency of integration, *in vivo*, was comparable to canonical *Hermes *gene vectors. These data demonstrate that some forms of episomal *Hermes *elements can efficiently reintegrate. The *in vitro *integration results with episomal *Hermes *elements are similar to those reported by Neiditch et al. who reported that RAG recombinase could cleave and transpose signal joints *in vitro *leading to the integration of signal joint episomes [14]. Overall these data further support the idea that V(D)J recombination and *hAT *elements may have evolved from a common ancestral transposable element.

Signal joints are no longer considered inert segments of DNA resulting from V(D)J recombination but are recognized as potential contributors to genome instability and disease in vertebrates [15]. Episomal forms of *hAT *may also contribute to a number of important aspects of the biology and natural history of these elements. *hAT *elements, like many other Class II transposable elements, have periodically undergone horizontal transfer[41]. While the exact mechanisms by which DNA is exchanged between species remain unknown, episomal forms of excised elements may provide a stable but integration-competent form of the element that can be more readily transferred between organisms. Interestingly, *Hermes *episomes were readily recovered from adult houseflies, a natural host of *Hermes*, indicating for the first time that these elements are actively transposing in this species and that they are likely to be active in somatic tissue. These conditions seem to be favorable for interspecies transfer and the data reported here show that some of these episomal forms of *Hermes *can undergo transposition. The presence of *hAT *episomes in unfertilized eggs suggests that these elements may also be maternally transmitted, a mechanism of transmission that has never been described for Class II transposable elements. This novel transmission mechanism might influence the transmission and population dynamics of these elements under certain conditions.

Finally, in addition to episomal *hAT *elements being transpositionally competent they also appeared to influence canonical element movement. The presence of episomal *Hermes *elements with 0 or 17 bp of intercalary DNA separating the terminal inverted repeats resulted in the reduced recovery of canonical *Hermes *transposition events *in vivo*. It is not clear at this time whether the effect is caused by repression or interference or some other mechanism. In germ-line transformation experiments involving the co-injection of canonical and episomal forms of *Hermes *elements the overall concentration of *Hermes *elements was comparable to that used in many insect transformation studies, suggesting that a simple titration effect was not responsible [42-44]. If, however, *Hermes *transposase binding to episomal forms of *Hermes *elements is different from binding to canonical forms then perhaps transposase titration is a possible mechanism. More experimentation is required to explore these interesting possibilities. Nonetheless, these data point to another possible biological role of episomal *hAT *elements, namely the regulation of element transposition.

## Conclusions

Episomal forms of the *hAT *elements *hobo *and *Hermes *are readily recovered under a variety of conditions. The recovery of episomal forms of *Hermes *from *M. domestica*, transgenic *D. melanogaster *and *A. aegypti *demonstrates the somatic activity of this element in these species. Episomal forms of *Hermes *are capable of participating in transposition/integration reactions *in vitro *and *in vivo*. They can also be transmitted maternally and under some conditions reduce the amount of canonical *Hermes *element transposition. These studies begin to reveal the potential biological significance of these widespread forms of extrachromosomal DNA.

## Methods

### *Musca domestica*

Adults were collected from natural populations (kindly provided by Dr. Elliott Krasfur, Iowa State University) and from laboratory colonies (the laboratory strain Cs was kindly provided by Dr. Jeffery Scott, Cornel University).

### *Drosophila melanogaster*

Using previously reported methods, transgenic lines were created using the *Hermes *gene vector 198H70-1 and the host strain *w*^1118 ^(Figure 1) [42]. The vector 198H70-1 contained, in addition to approximately 500 bp and 700 bp of the right and left ends of the *Hermes *transposable element, respectively [42], the transposase open reading frame under the regulatory control of the *D. melanogaster heat shock 70 *(*hsp70*) promoter and the Enhanced Green Fluorescent Protein (*EGFP*) open reading frame under the regulatory control of the *D. melanogaster Actin 5C *promoter [45], a kanamycin resistance gene, a ColE 1 origin of replication and the *E. coli *lac Z alpha peptide coding region [38]. Because these transgenic insects contained an autonomous (self-mobilizing) *Hermes *element, stable lines could not be established and maintained. Therefore transgenic populations were maintained by selecting EGFP-expressing individuals every other generation to ensure the transgene was at a high frequency within the laboratory population. The presence of an antibiotic resistance marker and a prokaryotic origin of replication enabled this vector to be used in plasmid rescue experiments from transgenic individuals.

Transgenic *D. melanogaster *(*w*^11118^) were also constructed using the *Hermes *gene vector 7011 [46]. This vector was identical to 198H70-1 except it lacked the kanamycin resistance gene, the origin of replication and the lacZ alpha peptide-coding region (Figure 1). Because this vector also was a self-mobilizing (autonomous) *Hermes *element stable lines could not be established and this line was also maintained by periodic selection of EGFP-expressing individuals, as described above.

*D. melanogaster *(*w*^11118^) were used as hosts to perform *Hermes *and *hobo *plasmid-based mobility assays in developing embryos.

### *Aedes aegypti*

Using reported methods the *Orlando *line (a wild-type laboratory strain) was transformed with the *Hermes *vector 198H70-1 [47]. Because of the low level of germ-line remobilization activity of *Hermes *in this species, maintenance did not require periodic selection for individuals expressing EGFP as was necessary for transgenic *D. melanogaster *containing the same vector [48].

### DNA Extractions

Genomic DNA was extracted from adult insects as described (using protocol 48 in "Drosophila: A laboratory manual", [49]) or using Wizard Genomic DNA Purification Kits (Promega, Madison Wisconsin) according to the manufacturers recommendations. Low molecular weight DNA (plasmids or episomes) was isolated according to the method of Hirt [50].

### Episome Analysis - Plasmid Rescue

One to five μg of undigested *D. melanogaster *genomic DNA was used in the electroporation of DH10-β *E. coli *(Invitrogen, Carlsbad, California) and the treated cells were grown in 1 ml of SOC [51] at 37°C for 1 hour. Cells were then concentrated by centrifugation, suspended in approximately 100 μl of SOC and spread on LB plates containing 25 μg/ml of kanamycin. Resistant colonies were isolated and plasmid DNA extracted and digested with restriction endonucleases *EcoR*I and *BamH*I. *Hermes *episomes originating from 198H70-1 excision are predicted to yield *Bam*H1 and *Eco*R1 fragments approximately 2.7, 2.6, 1.3, 0.6 and 0.4 kb in length depending on the exact structure of the joined ends of the element. Final confirmation of the structure of the recovered episomes was made by DNA sequencing using primers HL216 (5' GCA GGC GAC TGA GTA ACA ACA AT AAC AAC 3') and HR2500 (5' CAA TGA GTA TAC AAC ACA ACA AAG AAG TGA G 3').

### Episome Analysis 1

Using approximately 1 μg of *D. melanogaster *or *Ae. aegypti *(each transgenic with *Hermes *vector *198H70*-1) genomic DNA as a template, PCR was performed in 1× *Taq *polymerase buffer containing 2.5 mM MgCl_2_, 0.8 mM dNTPs and 8 pmoles each of primers HL216 and HR2500. Following an initial step of 95°C for 3 min. 25 iterations of the following cycle were performed: 95°C/15 sec, 55°C/15 sec, 72°C/30 sec. To complete the reaction and insure that all products were fully double-stranded the mixture was incubated at 72°C for 5 min. Episomal *Hermes *elements are expected to yield a 470 bp reaction product if the left and right inverted terminal repeats were precisely joined end to end. PCR amplification products were purified using QIAquick PCR Purification Kit (Qiagen, Valencia, CA) and ligated to the plasmid pGEM-T Easy (Promega). Electroporation was used to introduce some of the plasmids into DH10-β by and the DNA sequences of the plasmid inserts was determined from the recovered clones.

### Episome Analysis 2

This semi-nested-primer method was used to detect and analyze *Hermes *episomes in wild-type *M. domestica *and *D. melanogaster *(transgenic with *Hermes *vector 7011). The first reaction was performed in a volume of 50 μl with 1 mM dNTP's, 2.5 mM MgCl_2 _with primers Hermes2432F (5' AAT ATA CTT ATG CTC TTT TCC 3') (for *M. domestica*) or Hermes2681F (AAA ATA CTT GCA CTC AAA AGG C 3') (for *D. melanogaster*) and Hermes333R (5' TCG GAA CAT TTT GCT GTG 3'), each at 0.6 μM and 5% of the genomic DNA from individual insects as template. The reaction conditions involved a preliminary denaturation step of 95°C for 3 minutes followed by 25 cycles of 95°C/15 sec, 56°C/1 min, 72°C/1 min. Following these 25 cycles the reactions were incubated 72°C for 5 minutes. The reaction conditions for the second reaction were identical to those of the initial reaction but primers Hermes2432F (for *M. domestica*) or Hermes2681F (for *D. melanogaster*) and Cy5-66R+ (5' Cy5-AAT GAA TTT TTT GTT CAA GTG GCA AAG CAC 3') were used with 5 μl of a 20× dilution of the initial reaction as template. Following the reaction, approximately 5 μl was size-fractionated using a high resolution electrophoresis system consisting of a 1 mm thick 6% polyacrylamide in Tris Borate buffer and 8 M urea. The resulting gel was dried onto filter paper and scanned using the 633 nM light source of a Storm 860 gel/blot imaging system (Molecular Dynamics, Piscataway, NJ) to directly visualize the Cy5-labelled PCR products.

### Transposable Element Display

Transposable element display permits all members of a transposable element family in the genomic DNA of individual insects to be detected and visualized as unique PCR products. *Hermes *transposable element display was performed here as described [46].

### In vivo *hobo *Excision Assay

Pre-blastoderm *D. melanogaster *embryos (*w*^1118^) were injected with a mixture of the plasmids pHobo8bpdrKanOriLacZ and pHspHobo (each plasmid was at a concentration of 250 μg/ml) as described [52]. Injected embryos were incubated at 25°C overnight and approximately 18 hrs post injection the embryos were placed at 37°C for 1 hr and allowed to recover for 1 hr at 25°C. After heat shock and recovery viable embryos were collected and low molecular weight DNA was recovered as described [50]. To recover episomes low molecular weight DNA was digested with restriction endonucleases *Kpn *I and *Xba *I and used to electroporate *E. coli *(DH 10β). *Kpn *I and *Xba *I cut pHobo8bpdrKanOriLacZ within the donor plasmid backbone but not within the *hobo *element. Any excised episomal forms of *hobo *present within the sample will be resistant to digestion and will transform *E. coli *to kanamycin resistance with β-galactosidase activity. Plasmids from *E. coli *transformants were confirmed as *hobo *episomes by digesting with the restriction endonuclease *Sal *I and finally by determining the sequence of DNA spanning the inverted terminal repeats.

To recover *hobo *excision events (empty donor sites) recovered plasmids were introduced directly into *E. coli *and selected for chloramphenicol resistance. Chloramphenicol resistant colonies that were negative for β-galactosidase activity and sensitive to kanamycin were confirmed as excision products by digestion with restriction endonuclease digestion with *Kpn *I and *Xba *I.

### *In vitro Hermes *Episome Transposition Assay

*In vivo *inter-plasmid transpositions assays have been used extensively to investigate insect *hAT *elements (e.g. Sarkar et al. [38]). Here the assay has been adapted to a cell-free system and consists of a donor element (episomal forms of *Hermes*), a target plasmid (pGDV1) and purified *Hermes *transposase [53]. The episomal forms of *Hermes *tested in this assay contained a kanamycin resistance gene, a ColE1 origin of replication and the *E. coli lacZ *alpha-peptide coding region [38]. The target plasmid, pGDV1, is a gram-positive chloramphenicol resistant plasmid that cannot replicate in *E. coli *without the addition of a species-compatible origin of replication [38,54]. Reactions were performed in 20 μl of 20 mM HEPES pH7.9, 25% glycerol, 5 mM MgCl_2_, 4 μg bovine serum albumin, 2 mM DTT with 250 ng each of donor and target plasmids, ands 1 μg of purified *Hermes *transposase [9]. The reaction was incubated at 30°C for 1-2 hrs at which time 80 μl of stop solution (50 mM Tris pH 7.5, 0.5 mg/ml proteinase K, 10 mM EDTA and 0.1 μg/ml tRNA) was added and incubated at 37°C for 1 hour. The reaction was extracted with phenol/chloroform and the DNA was precipitated with sodium acetate and ethanol and the dried precipitate was dissolved in 20 μl of water. Fifteen microliters were introduced into 80 μl of *E. coli *(DH10-β) by electroporation. Following electroporation 900 μl of SOC was added and the cells were incubated at 37°C for 1 hour at which time 1 μl was plated on LB plates containing ampicilin (50 μg/ml) and X-gal (20 μg/ml) to assess plasmid recovery. The rest of the cells were divided and plated on 10 LB plates containing chloramphenicol (10 μg/ml), kanamycin (25 μg/ml) and X-gal (20 μg/ml) to select for donor transpositions into the target plasmid. Restriction mapping and DNA sequencing of putative transposition events confirmed the presence and of transposed *Hermes *elements.

### *In vivo Hermes *Episome Transposition Assay

Germ-line integration was used to assess the mobility properties of episomal forms of *Hermes in vivo*. A 1 kb fragment containing the coding region of the DsRed protein under the regulatory control of the 3× P3 promoter was inserted between the terminal sequences of episomal forms of *Hermes *that contained a kanamycin resistance gene, a plasmid origin of replication and the LacZ alpha peptide coding region. *Hermes *transformation vectors were created with episomal forms containing 0, 17, 34, 80 and 160 bp of intercalary DNA separating the terminal inverted repeats of the element. The *Hermes *gene vectors HermesActin5CEGFP and Hermes3 × P3EGFP were used as controls [45,55]. *Hermes *transposase was supplied by co-injecting the plasmid pHSHH1.9 along with the vector containing plasmids being tested using methods previously described [42].

Transgenic insects were subsequently analyzed by transposable element display to confirm the presence of *Hermes*. Integration sites were sequenced following isolation, re-amplification and sequencing of transposable element display bands as described [46]. The DNA sequence flanking the integrated *Hermes *elements were used in a BLAST search [56] of the *D. melanogaster *genome sequence in FlyBase [57]. A sequence logo [58] was created using the aligned 8 bp of DNA immediately flanking the left inverted repeat of each integrated *Hermes *element using WebLogo [59]

## Authors' contributions

DAO'B performed the experiments involving *hobo*, the analysis of episomes in *M. domestica*, *in vivo *analysis of episomal *Hermes *element transposition in *D. melanogaster*. He compiled the data and wrote the manuscript. CDS performed the analysis of *Hermes *episomes in transgenic *D. melanogaster*. KP performed the *in vitro *analysis of episomal *Hermes *elements. RAS performed the *in vitro *analysis of episomal *Hermes *elements and assisted in data analysis. RHH developed the *in vitro *transposition assays for *Hermes *elements. PWA supervised CDS and RHH, developed the *in vitro *transposition assays for *Hermes *elements, performed the analysis of *Hermes *episomes in transgenic *D. melanogaster*, analyzed data and wrote the manuscript. All authors have read and approved the final manuscript.

## Author Information

RA Subramanian is currently at SUNY Downstate Medical Center; Department of Emergency Medicine; 450 Clarkson Avenue; Brooklyn, NY 11203, USA.

CD Hoddle (nee Stosic) is currently at the Department of Entomology, University of California, Riverside, CA 92521, USA.
